# Myostatin-deficiency in mice increases global gene expression at the Dlk1-Dio3 locus in the skeletal muscle

**DOI:** 10.18632/oncotarget.13966

**Published:** 2016-12-15

**Authors:** Keisuke Hitachi, Kunihiro Tsuchida

**Affiliations:** ^1^ Division for Therapies Against Intractable Diseases, Institute for Comprehensive Medical Science (ICMS), Fujita Health University, Toyoake, Aichi 470-1192, Japan

**Keywords:** myostatin, miRNA, callipyge, IG-DMR ncRNA, skeletal muscle

## Abstract

Myostatin, a member of the transforming growth factor-beta superfamily, is a negative regulator of skeletal muscle growth and development. Myostatin inhibition leads to increased skeletal muscle mass in mammals; hence, myostatin is considered a potential therapeutic target for skeletal muscle wasting. However, downstream molecules of myostatin in the skeletal muscle have not been fully elucidated. Here, we identified the Dlk1-Dio3 locus at the mouse chromosome 12qF1, also called as the *callipyge* locus in sheep, as a novel downstream target of myostatin. In skeletal muscle of myostatin knockout mice, the expression of mature miRNAs at the Dlk1-Dio3 locus was significantly increased. The increased miRNA levels are caused by the transcriptional activation of the Dlk1-Dio3 locus, because a significant increase in the primary miRNA transcript was observed in myostatin knockout mice. In addition, we found increased expression of coding and non-coding genes (*Dlk1*, *Gtl2*, *Rtl1/Rtl1as*, and *Rian*) at the Dlk1-Dio3 locus in myostatin-deficient skeletal muscle. Moreover, epigenetic changes, associated with the regulation of the Dlk1-Dio3 locus, were observed in myostatin knockout mice. Taken together, this is the first report demonstrating the role of myostatin in regulating the Dlk1-Dio3 (the *callipyge*) locus in the skeletal muscle.

## INTRODUCTION

Myostatin, a cytokine belonging to the transforming growth factor-beta superfamily, negatively regulates skeletal muscle growth and development through the regulation of anabolic and catabolic pathways in skeletal muscles [1]. Systemic administration of myostatin results in decreased skeletal muscle mass in mice [2], whereas *myostatin*-null mice exhibit 200–300% increase in skeletal muscle weight [3]. In human, a mutation that may be related to mis-splicing in the *myostatin* gene was found in a child with muscle hypertrophy [4]. The loss of skeletal muscle mass is a clinically important problem in diseases (cachexia) and aging (sarcopenia). Thus, myostatin is a promising therapeutic target for skeletal muscle wasting due to several diseases and aging; however, its downstream molecules in the skeletal muscle have not been fully identified.

The *callipyge* (*CLPG*) phenotype in sheep shows postnatal skeletal muscle hypertrophy of the lower body [5]. A point mutation (A to G transition) on the regulatory element located between *Dlk1* and *Gtl2* (also called *Meg3*) genes is associated with the *callipyge* phenotype [6]. The paternally inherited *CLPG* mutation can induce skeletal muscle hypertrophy with an increase in *Dlk1* and *Rtl1* (also known as *PEG11*) expression [7, 8]. Contrary to the paternally inherited *CLPG* mutation, the maternally inherited *CLPG* mutation decreases the expression levels of the paternally expressed genes with an increase in *Gtl2*, *antiRtl1* (*Rtl1as*), and *Rian/Meg8* expression [7, 8]. In addition, the maternally inherited *CLPG* mutation upregulates the expression of a miRNA cluster containing more than 100 miRNAs, located at the Dlk1-Dio3 locus [9]. Previous reports have indicated the roles of these miRNAs in skeletal muscle regeneration and mitochondrial biogenesis [10, 11]. Although the transcription factor MEF2a was shown to be involved in regulating the expression of this miRNA cluster in skeletal muscle regeneration [10], the precise mechanism that controls the transcription of the Dlk1-Dio3 locus in skeletal muscle remains unclear.

We have previously examined the global miRNA expression profile in the skeletal muscle of myostatin knockout mice by microarray analysis and found 11 miRNAs whose expression was upregulated or downregulated in myostatin-deficient skeletal muscle [12]. We showed that one of these miRNAs, miR-486, is a direct molecular target of myostatin in the regulation of skeletal muscle mass [12]. However, the role of other miRNAs whose expression was altered in myostatin knockout mice remains to be determined. To characterize whether these miRNAs are associated with the muscular phenotype in myostatin knockout mice, here we validated the miRNA expression and found the increased expression of the miRNA cluster at the Dlk1-Dio3 locus in myostatin-deficient skeletal muscle. In addition to miRNAs, myostatin deficiency also increased the expression of a large number of genes located within the Dlk1-Dio3 locus in the skeletal muscle. Moreover, we found epigenetic changes, decreased DNA methylation at the Dlk1-Dio3 locus and increased IG-DMR ncRNA expression, in myostatin knockout mice. Taken together, the Dlk1-Dio3 locus was shown to be a new downstream target of myostatin signaling, suggesting that myostatin deficiency leads to activation of transcription at the Dlk1-Dio3 locus *via* the regulation of epigenetic modulation.

## RESULTS

### Increased expression of miRNAs encoded by the Dlk1-Dio3 locus in myostatin-deficient skeletal muscle

**Figure 1:**
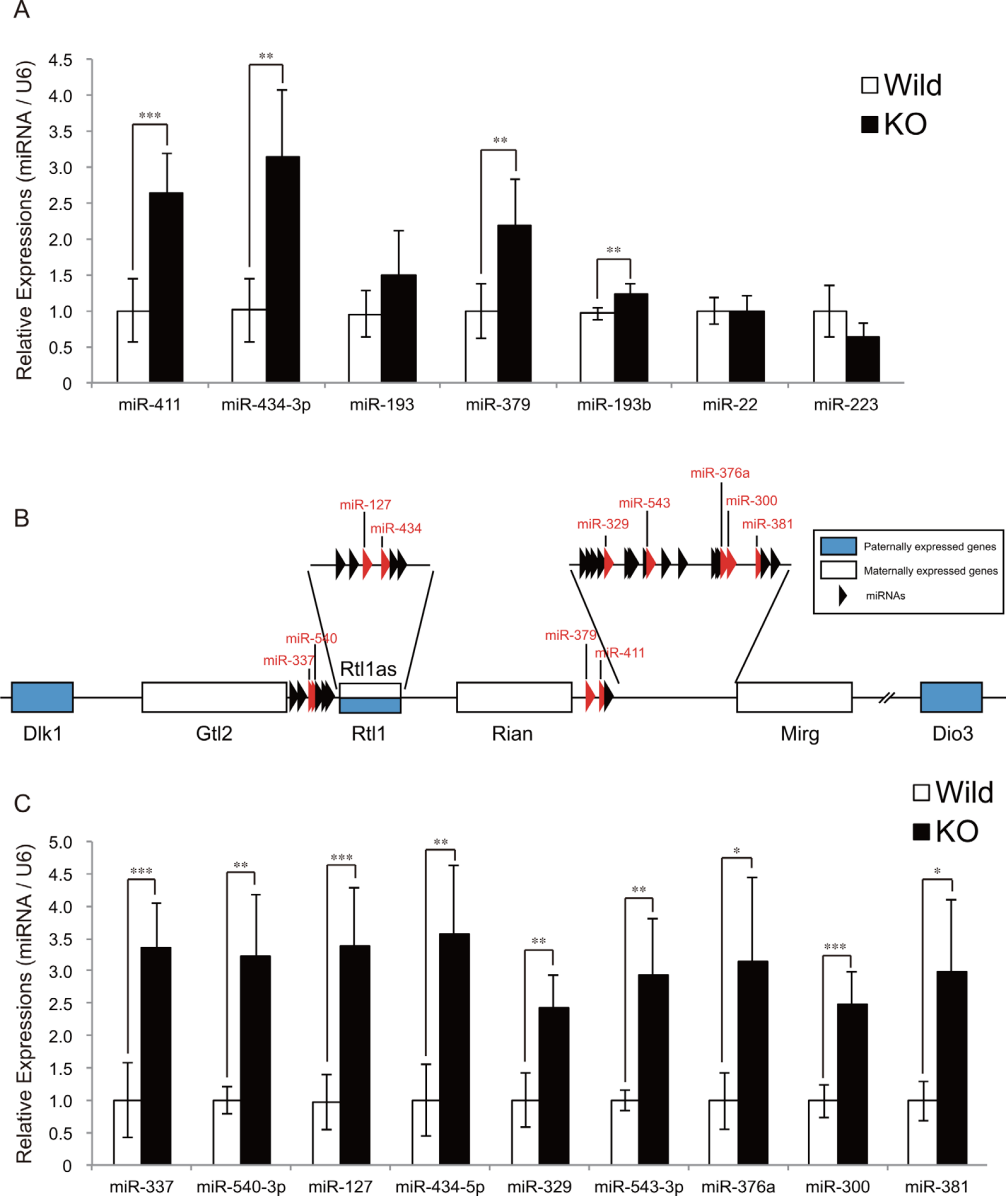
The expression of miRNAs derived from the Dlk1-Dio3 locus is upregulated in myostatin-deficient skeletal muscle (**A**) A significant increase in miR-411, miR-434-3p, miR-379, and miR-193b expression in myostatin-deficient skeletal muscle at 13 weeks of age was determined by quantitative RT-PCR. (**B**) Schematic diagram of the Dlk1-Dio3 locus structure. This locus contains imprinting genes; *Dlk1*, *Rtl1*, and *Dio3* (shown as blue boxes) are paternally expressed, whereas *Gtl2*, *Rtl1as*, *Rian*, *Mirg* (shown as white boxes), and miRNAs (arrowheads) are maternally expressed. Red arrowheads indicate the miRNAs whose expression was validated by qRT-PCR in Figure 1C. (**C**) Quantitative RT-PCR analysis shows a significant increase in the expression of miR-337, miR-540-3p, miR-127, miR-434-5p, miR-329, miR-543-3p, miR-376a, miR-300, and miR-381 in myostatin-deficient skeletal muscle at 13 weeks of age. The wild-type mice are the same age as the myostatin knockout mice. The results were normalized to *U6* small RNA expression. Data are the mean ± SD (*n* = 5). *P < 0.05. **P < 0.01. ***P < 0.001.

We previously identified 11 miRNAs which were upregulated or downregulated in myostatin knockout mice by microarray analysis, and validated the expression of miR-486 and miR-206 [12]. However, the expression of the remaining 9 miRNAs (miR-411, miR-434-3p, miR-299*, miR-193, miR-146b, miR-379, miR-193b, miR-22, and miR-223) has not been verified. To reveal the role of these miRNAs in the muscular phenotype of myostatin knockout mice, we quantified their expression by qRT-PCR, and observed a significant increase in the expression of miR-411, miR-434-3p, miR-379, and miR-193b in myostatin knockout mice (Figure 1A). Among these, the increased expressions of miR-411, miR-434-3p, and miR-379 were of interest because these miRNAs are expressed from the mouse chromosome 12qF1 (chr12qF1), called as the Dlk1-Dio3 locus (Figure 1B). The Dlk1-Dio3 locus is highly conserved in mammals and contains both paternally and maternally expressed genes [13]. In addition, the miRNA cluster (more than 100 miRNAs) is maternally transcribed from the Dlk1-Dio3 locus [14]. We randomly picked 9 miRNAs (miR-337, miR-540-3p, miR-127, miR-434-5p, miR-329, miR-543-3p, miR-376a, miR-300, and miR-381) expressed from the Dlk1-Dio3 locus and validated their expression by qRT-PCR. The results of this quantification showed that the expression of all miRNAs tested was significantly increased in myostatin knockout mice (Figure 1C). These results indicate that myostatin deficiency increases the expression of the miRNA cluster located in the Dlk1-Dio3 locus in the skeletal muscle.

**Figure 2:**
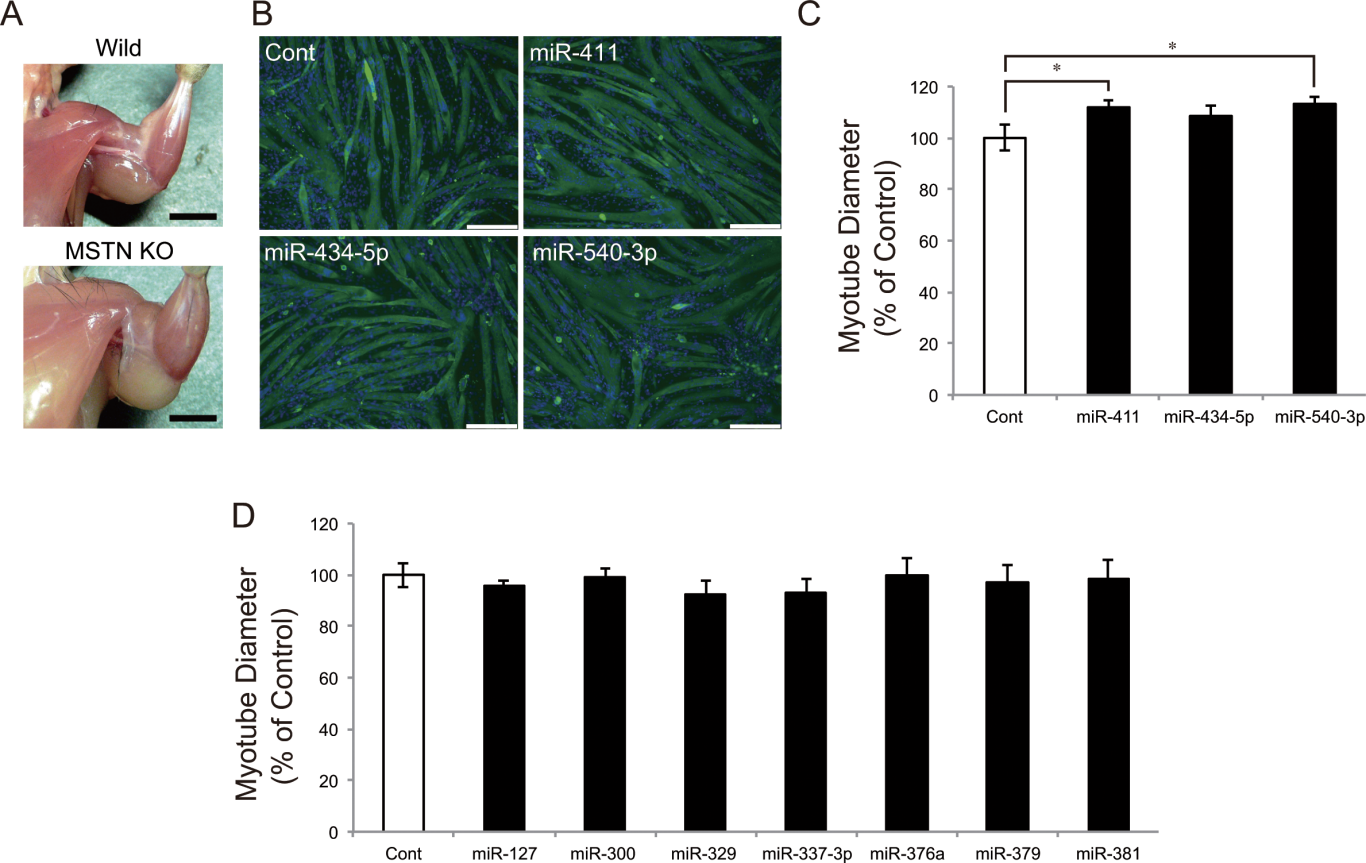
miR-411 and miR-540-3p increase the C2C12 myotube diameters (**A**) Myostatin knockout mice show a significant increase in the skeletal muscle mass. (**B**) Confluent C2C12 cells were differentiated into myotubes and transfected with 25 nM of miRNA mimics. Myotubes were transfected with miR-411, miR-434-5p, and miR-540-3p mimics and immunostained with an anti-myosin heavy chain antibody and DAPI. (**C** and **D**) Myotube diameter was measured as described in the materials and methods section and was shown as the percent of the control. Transfection of miR-411 and miR-540-3p mimics, but not other miRNAs, significantly increased the myotube diameter. Bar, 250 μm. Error bars indicate the SD of 3 independent experiments. *P < 0.05.

Myostatin deficiency leads to remarkable skeletal muscle hypertrophy in mice (Figure 2A). To clarify the role of these miRNAs in skeletal muscle hypertrophy induced by myostatin deficiency, we transfected miRNA mimics (miR-127, miR-300, miR-329, miR-337-3p, miR-376a, miR-379, miR-381, miR-411, miR-434-5p, and miR-540-3p) into C2C12 myotubes and measured the myotube diameter. Immunofluorescence staining of myotubes with an antibody specific to the myosin heavy chain, which is a terminally differentiated marker of skeletal muscle cells, demonstrated that overexpression of miR-411 and miR-540-3p significantly increased the C2C12 myotube diameter (miR-411; 100 ± 4.89 *vs*. 112 ± 2.94, *P* = 0.022, and miR-540-3p; 100 ± 4.89 *vs*. 113 ± 3.02, *P* = 0.017) (Figure 2B and 2C). Overexpression of miR-434-5p tended to increase the myotube diameter (100 ± 4.89 *vs*. 108 ± 4.23, *P* = 0.091), whereas overexpression of the remaining 7 miRNAs (miR-127, miR-300, miR-329, miR-337-3p, miR-376a, miR-379, and miR-381) showed no significant effect on myotube diameter (Figure 2D).

**Figure 3:**
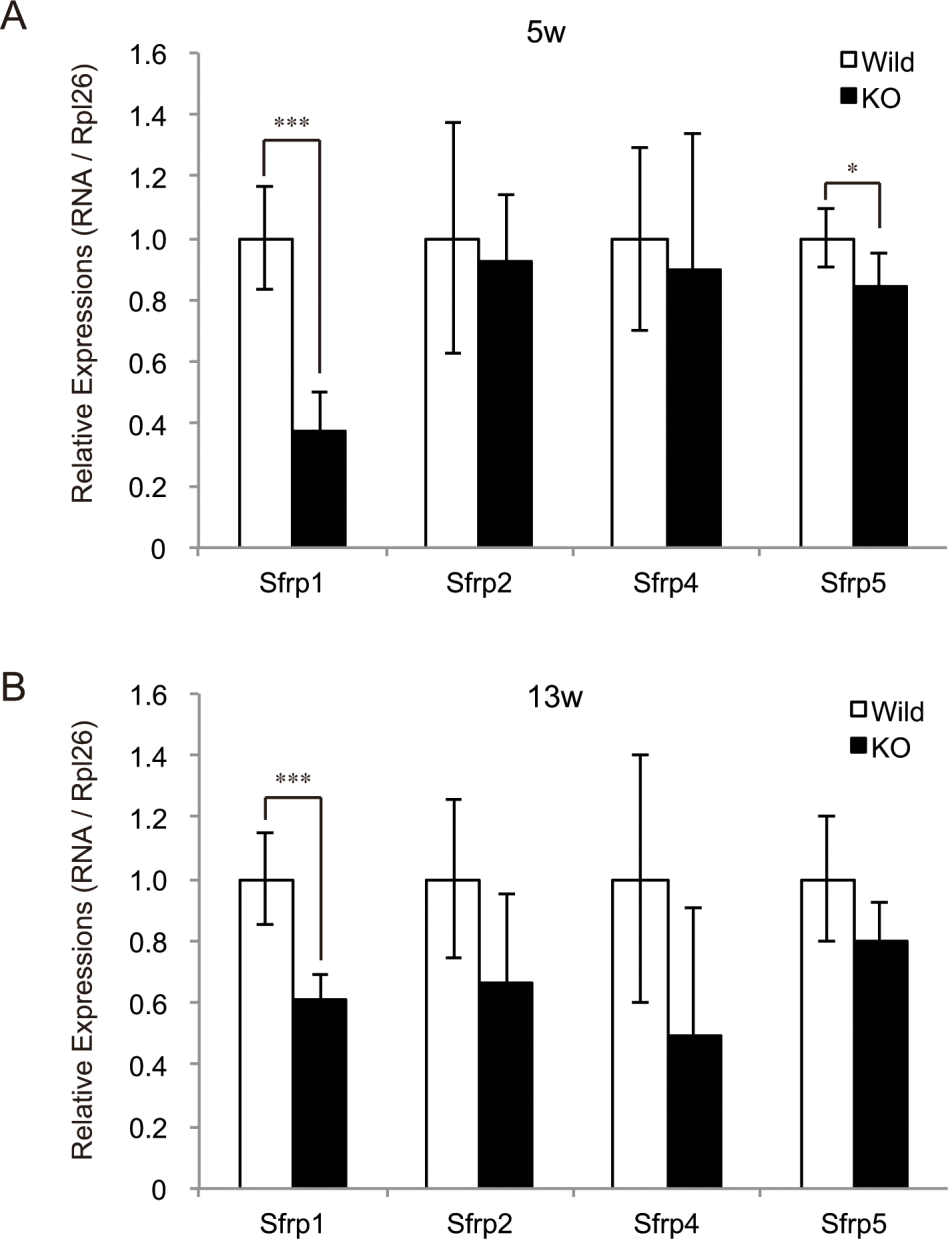
Decreased expression of SFRP family genes in myostatin-deficient skeletal muscle Quantification of expression levels of *SFRP* (Secreted frizzled-related protein) genes, which are secreted inhibitors of Wnt signaling pathway, was performed by qRT-PCR. A significant decrease in the expression of *Sfrp1* was observed in myostatin-deficient skeletal muscle at 5 (**A**) and 13 (**B**) weeks of age. In addition, a significant decrease in *Sfrp5* expression was also detected in myostatin knockout mice at 5 weeks of age (A). The wild-type mice are the same age as the myostatin knockout mice. The results were normalized to the expression of *Rpl26*. Data are mean ± SD (*n* = 5). *P < 0.05, ***P < 0.001.

A previous study has revealed that the miRNA cluster at the Dlk1-Dio3 locus targets *SFRP* genes, which encode secreted inhibitors of the Wnt signaling pathway, in murine skeletal muscle [10]. In addition, myostatin was shown to negatively regulate skeletal muscle growth and development through the downregulation of Wnt signaling pathway [15–17]. We therefore evaluated the expression levels of *SFRP* genes (*Sfrp1*, *Sfrp2*, *Sfrp4*, and *Sfrp5*) in myostatin-deficient skeletal muscle by qRT-PCR. A significant decrease in *Sfrp1* expression was observed in both 5- and 13-week-old myostatin knockout mice (Figure 3A and 3B). At 5 weeks of age, the expression level of *Sfrp5* was also significantly reduced in myostatin knockout mice. Taken together, these results suggest that increased expression of the miRNA cluster on chr12qF1 would contribute to decrease the expression of *Sfrp* family genes in myostatin knockout mice. This could explain one of the mechanisms for the muscular phenotype in myostatin knockout mice.

### Myostatin deficiency increased transcription at the Dlk1-Dio3 locus in skeletal muscle

**Figure 4:**
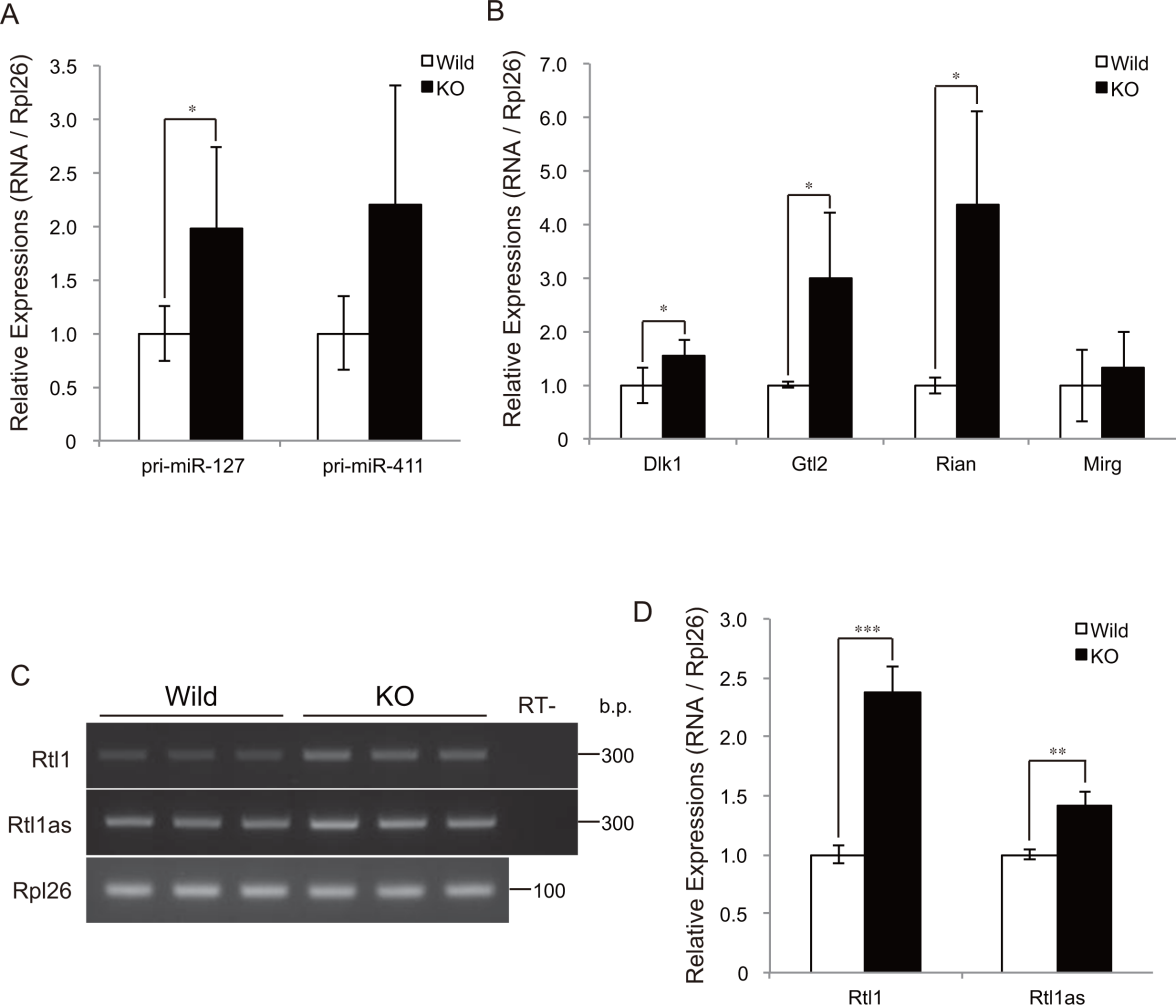
Myostatin deficiency increases transcription at the Dlk1-Dio3 locus (**A**) Quantitative RT-PCR analysis shows a significant increase in the expression of the primary transcript of miR-127 in myostatin-deficient skeletal muscle at 13 weeks of age. (**B**) Myostatin deficiency increases the expression levels of the maternally expressed *Gtl2* and *Rian*, as well as the paternally expressed *Dlk1*. The results were normalized to *Rpl26* expression. Data are the mean ± SD (*n* = 5). *P < 0.05. (**C**) Strand-specific RT-PCR shows increased expression of *Rtl1* and *Rtl1as* in myostatin-deficient skeletal muscle. (**D**) Quantitative analyses of *Rtl1* and *Rtl1as* expression using Image J software. The wild-type mice are the same age as the myostatin knockout mice. Data are the mean ± SD (*n* = 3). **P < 0.01, ***P < 0.001.

To determine whether myostatin deficiency activates the transcription of chr12qF1 miRNAs, we further quantified the expression levels of the primary transcripts of miR-127 (pri-miR-127) and miR-411 (pri-miR-411) by qRT-PCR. The quantification results showed a significant increase in pri-miR-127 expression in myostatin knockout mice (Figure 4A). The expression level of pri-miR-411 also tended to increase (Figure 4A). Besides miRNAs, the Dlk1-Dio3 locus includes paternally expressed genes (*Dlk1*, *Rtl1*, and *Dio3*) and maternally expressed genes (*Gtl2*, *Rtl1as*, *Rian*, and *Mirg*). This prompted us to quantify the expression levels of these chr12qF1 genes in myostatin-deficient skeletal muscle. Quantitative RT-PCR analysis showed that the expression levels of *Dlk1*, *Gtl2*, and *Rian*, but not *Mirg*, were significantly increased in myostatin knockout mice (Figure 4B). Strand-specific semi-quantitative RT-PCR analysis also showed a significant increase in *Rtl1* and *Rtl1as* expression (Figure 4C and 4D). These results indicate that myostatin deficiency increased the expression of chr12qF1 transcripts including miRNAs, regardless of the paternal or maternal chromosome.

**Figure 5:**
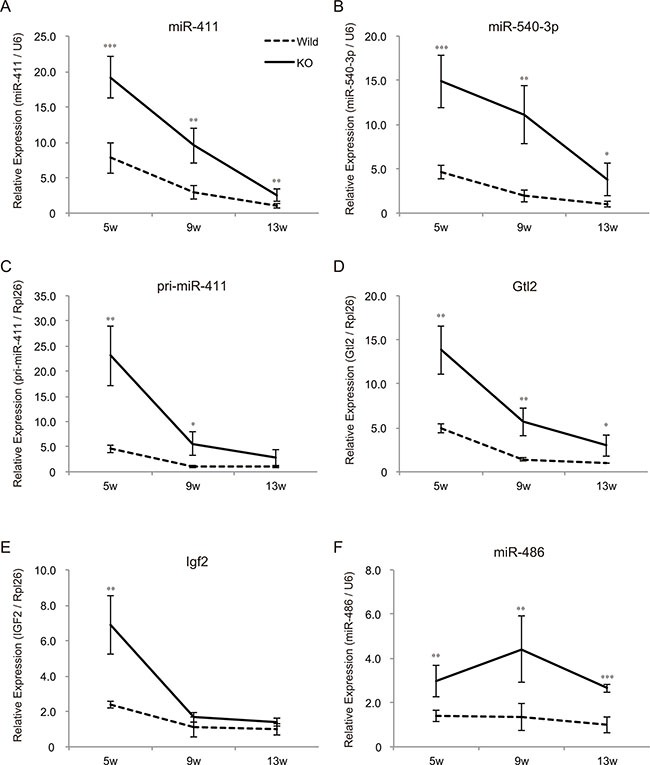
Mice growth decreases the expression levels of imprinting genes in the skeletal muscle Quantitative RT-PCR showing the decreased expression of miR-411 (**A**) miR-540-3p (**B**) pri-miR-411 (**C**) and *Gtl2* (**D**) in both wild-type and myostatin knockout mice with age. (**E**) The expression level of *Igf2*, the growth-related imprinting gene, is also decreased in the skeletal muscle as mice grow. (**F**) The expression of miR-486 that is increased in myostatin knockout mice is not changed with age. The expression level of either *U6* or *Rpl26* was used to normalize the qRT-PCR results. Data are the mean ± SD (*n* = 5). *P < 0.05, **P < 0.01, ***P < 0.001.

We have previously shown that skeletal muscle hypertrophy in myostatin knockout mice is initially observed at 5 weeks of age [12]. Consequently, we next evaluated the expression levels of chr12qF1 transcripts at 5, 9, and 13 weeks of age by qRT-PCR. Although the expression of chr12qF1 transcripts was higher in myostatin knockout mice compared to that in wild-type mice, the expression level of these transcripts was drastically decreased with increasing age (Figure 5A–5D). The same trend was observed for another imprinting gene, *Igf2*, but not in the non-imprinting miRNA, miR-486 (Figure 5E and 5F), suggesting that the expression of imprinting genes is gradually decreased in the skeletal muscle, as mice get older.

### Altered DNA methylation status and non-coding RNA expression in myostatin-deficient skeletal muscle

**Figure 6:**
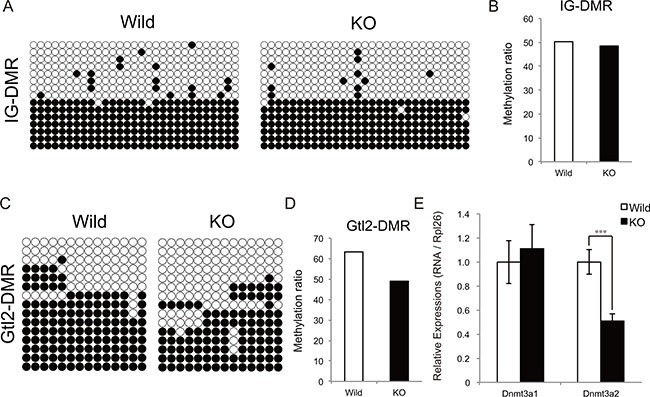
Methylation status of the IG-DMR and Gtl2-DMR domain in myostatin-deficient skeletal muscle Bisulfite sequencing analysis shows the methylation maps around the IG-DMR (**A**) and Gtl2-DMR (**C**) domain. The CpG sites are indicated by circles (black circles indicate methylated and white circles indicate unmethylated cytosine sites, respectively) and each row represents an individual clone. The methylation ratio of the IG-DMR (**B**) and Gtl2-DMR (**D**) domain in the myostatin knockout mice is shown as the percentage of that in wild-type mice. (**E**) Quantitative RT-PCR showing decreased *Dnmt3a2* expression in myostatin-deficient skeletal muscle. The wild-type mice are the same age as the myostatin knockout mice. The results were normalized to the expression of *Rpl26*. Data are the mean ± SD (*n* = 5). ***P < 0.001.

The DNA methylation levels of two differentially methylated regions (IG-DMR and Gtl2-DMR) located between the *Dlk1* and *Gtl2* genes affect the expression of chr12qF1 transcripts [18–20]. We therefore examined whether the increased expression of genes at the Dlk1-Dio3 locus in myostatin knockout mice depends on the changes in DNA methylation status using bisulfite sequencing. Although the DNA methylation status of IG-DMR was not changed (Figure 6A and 6B), that of Gtl2-DMR was found to be decreased in myostatin knockout mice (Figure 6C and 6D). We further investigated whether myostatin deficiency affects the expression of *de novo* DNA methyltransferases (Dnmt3a1 and Dnmt3a2) in the skeletal muscle by qRT-PCR and found that the expression of *Dnmt3a2* but not *Dnmt3a1* was significantly decreased in myostatin knockout mice (Figure 6E).

**Figure 7:**
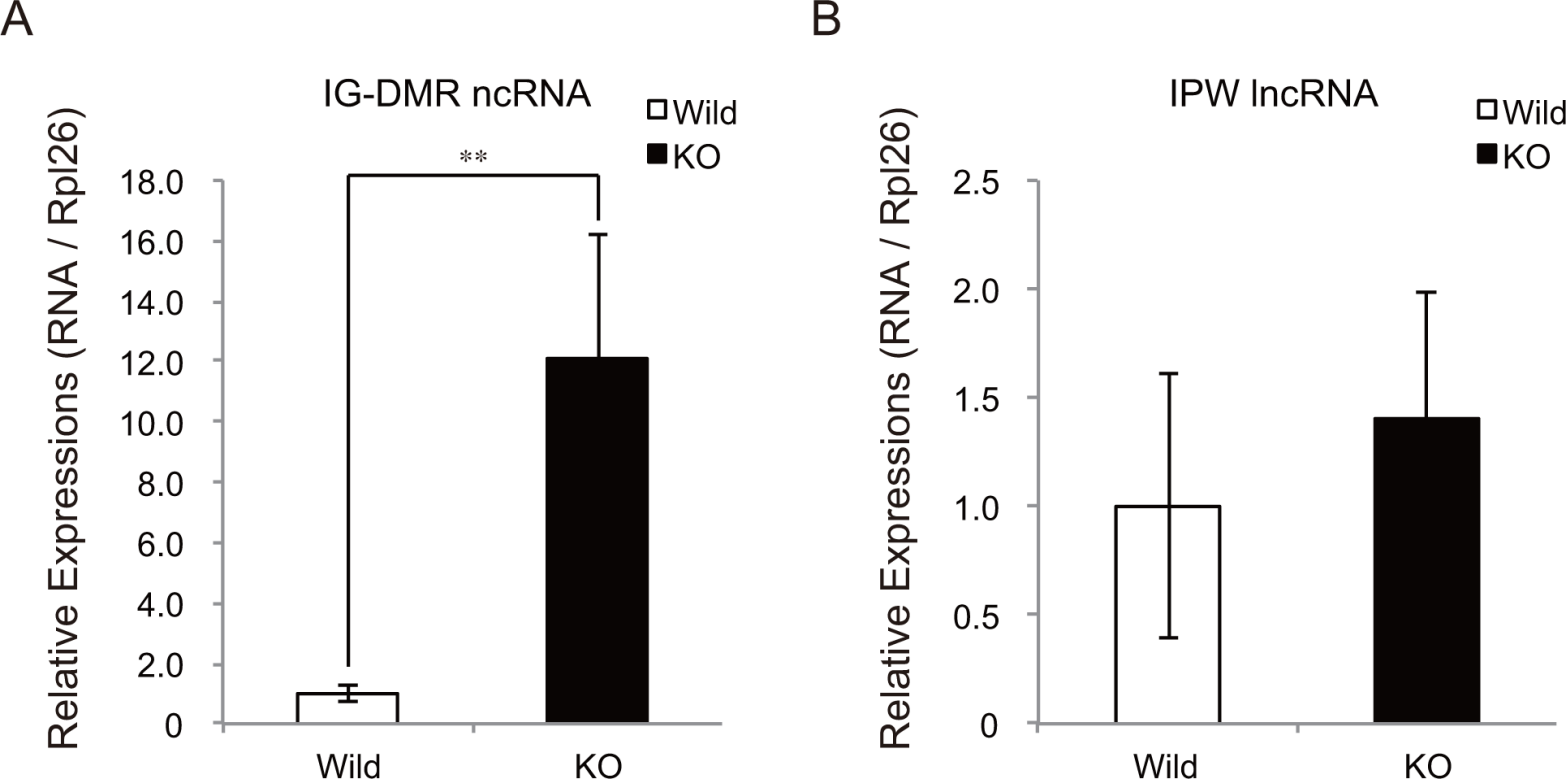
Increased expression of IG-DMR ncRNA in myostatin-deficient skeletal muscle (**A**) Quantitative RT-PCR analysis shows the increased expression of IG-DMR ncRNA in myostatin-deficient skeletal muscle at 13 weeks of age. (**B**) Myostatin deficiency does not affect the expression level of IPW lncRNA. The wild-type mice are the same age as the myostatin knockout mice. The results were normalized to the expression of *Rpl26*. Data are the mean ± SD (*n* = 5). **P < 0.01.

Recently, IG-DMR and IPW RNAs were identified to regulate the expression of almost all the transcripts at the Dlk1-Dio3 locus [21, 22]. IG-DMR RNA is an enhancer RNA-like ncRNA expressed from the IG-DMR domain at the Dlk1-Dio3 locus. IPW RNA is a paternally expressed long ncRNA (lncRNA) located in the Prader-Willi syndrome-associated locus. Notably, loss-of-function of IG-DMR ncRNA and gain-of-function of IPW lncRNA resulted in a drastic decrease in chr12qF1 transcripts in mouse ESCs and human iPSCs, respectively. We thus examined the expressions of these ncRNAs in myostatin knockout mice. Intriguingly, qRT-PCR analysis showed a drastic increase in IG-DMR ncRNA expression in myostatin knockout mice (Figure 7A), whereas the expression level of IPW lncRNA was not changed (Figure 7B). From these results, we concluded that myostatin deficiency affected the expression of epigenetic modulators including DNA methyltransferase and ncRNA in the skeletal muscle.

## DISCUSSION

Myostatin is a powerful negative regulator of skeletal muscle growth and development. Although myostatin inhibition in the skeletal muscle is valuable for medical and agricultural applications, its downstream molecules in the skeletal muscle have not been fully identified. In this study, we found higher expression of mature miRNAs derived from the mouse chromosome 12qF1, called as the Dlk1-Dio3 locus, in the tibialis anterior muscle of myostatin knockout mice compared to that of wild-type mice. Our results are consistent with a recent study by Wu et al., wherein the expression of miR-431 derived from the Dlk1-Dio3 locus is increased in myostatin knockout mice [23]. In addition to the miRNAs, we also found increased expression levels of both coding and non-coding genes at the Dlk1-Dio3 locus, regardless of paternal or maternal origin, in myostatin-deficient skeletal muscle. These observations indicate that myostatin deficiency leads to activate the transcription at the Dlk1-Dio3 locus. In fact, we showed that an increase in mature miR-127 expression is the result of activated transcription of the primary miR-127 transcript in myostatin knockout mice. Notably, the genes at the Dlk1-Dio3 locus are transcribed as one long polycistronic RNA in the skeletal muscle [24]. Taken together, our study is the first to show that myostatin deficiency increases the global gene expression at the Dlk1-Dio3 locus in murine skeletal muscle.

In sheep, the Dlk1-Dio3 locus is associated with the double-muscle phenotype called *callipyge*, which shows a 30–40% postnatal increase in the mass of hindlimb muscles [5]. A paternally inherited *callipyge* mutation is responsible for an increase in *Dlk1* and *Rtl1* expression [6]. Transgenic overexpression of *Dlk1* or *Rtl1* in mice resulted in increased skeletal muscle mass [25, 26], whereas *Dlk1* ablation resulted in reduced body weight and skeletal muscle mass [27], indicating that *Dlk1* and *Rtl1* are responsible for muscular hypertrophy phenotype in the *callipyge* sheep. Intriguingly, bi-allelic mutation of this locus is not characterized by muscle hypertrophy, because increased expression of maternally expressed ncRNAs might inhibit the Dlk1 expression [25]. Most recently, Gao *et al*. showed that deletion of the maternally expressed miR-379/miR-544 cluster located between the *Rian* and *Mirg* genes resulted in *callipyge*-like muscular hypertrophy with increased Dlk1 expression in mice [28]. Thus, increased expression of miRNAs at this locus is likely to attenuate muscular hypertrophy phenotype caused by Dlk1 overexpression. We did find a small but a significant increase in *Dlk1* expression and a remarkable increase in *Rtl1* expression in the myostatin knockout mice. Concomitantly, a significant increase in maternally expressed miRNAs at the Dlk1-Dio3 locus was also observed. Although we demonstrated the potency of the Dlk1-Dio3 locus derived miRNAs (miR-411 and miR-540-3p) to induce myotube hypertrophy using an *in vitro* model, muscular hypertrophy phenotype observed in the *callipyge* mutation and myostatin deficiency would be caused by slightly different mechanism. In fact, a remarkable increase in skeletal muscle mass in myostatin knockout mice was observed after 5 weeks of age [12], whereas muscle hypertrophy was not observed in mice with the deletion of the miR-379/miR-544 cluster after 3 weeks of age [28]. In addition to the Dlk1-Dio3 locus, myostatin signaling was shown to regulate the expression of miR-486 [12], miR-29b/c [29], and E3 ubiquitin ligase Atrogin-1/MAFbx [30] in skeletal muscle. Thus, in spite of an increase in miRNAs on the Dlk1-Dio3 locus, the increased expression of *Dlk1* and *Rtl1* might be sufficient to induce the muscular hypertrophy in myostatin knockout mice. Alternatively, increased expression of miRNAs at the Dlk1-Dio3 locus is likely to contribute to the promotion of muscle regeneration rather than the skeletal muscle hypertrophy in myostatin knockout mice. Snyder *et al*. showed that the expression of miRNAs from the Dlk1-Dio3 locus is downregulated in *Mef2a* knockout mice and a shortage of these miRNAs results in upregulation of the *SFRP* genes that impair skeletal muscle regeneration by attenuating Wnt signaling [10]. Consistent with a previous report [15], we observed significantly decreased expression of *SFRP* genes in myostatin-deficient skeletal muscle in parallel with increased expression of miRNAs at the Dlk1-Dio3 locus (Figures 1 and 3). Considering the fact that myostatin inhibition facilitates muscle regeneration [31], increased expression of miRNAs at the Dlk1-Dio3 locus in myostatin-deficient skeletal muscle might contribute to the promotion of muscle regeneration in addition to inducing muscular hypertrophy.

In this study, myostatin deficiency was shown to affect the expression of epigenetic modulators; *Dnmt3a2* and IG-DMR ncRNA. Dnmt3a2 is one of the *de novo* DNA methyltransferases in humans and mice [32], and reduced *Dnmt3a2* expression causes hypomethylation of imprinted genes in mouse ESCs [33]. Transcription from the Dlk1-Dio3 locus is controlled by Gtl2-DMR that is hypomethylated in myostatin knockout mice. Thus, increased expression of the Dlk1-Dio3 locus in myostatin-deficient skeletal muscle would be a result of a decrease in *Dnmt3a2* expression. It is also worthwhile to note that we used purchased wild-type mice as a control, therefore, it is possible that the differences in diet and environment would contribute to the different DNA methylation status at the Gtl2-DMR. In addition to *Dnmt3a2*, we observed greater than 10-fold increase in IG-DMR ncRNA expression in myostatin-deficient skeletal muscle. IG-DMR ncRNA is produced from the IG-DMR domain at the Dlk1-Dio3 locus and the knockdown of the IG-DMR ncRNA drastically reduces the expression of both paternally and maternally expressed genes at the Dlk1-Dio3 locus in ESCs [21]. Although whether IG-DMR ncRNA is required for activation of the Dlk1-Dio3 locus in the skeletal muscle remains to be determined, it seems likely that increased expression of IG-DMR ncRNA would be one of the factors that activates the expression at the Dlk1-Dio3 locus in myostatin knockout mice.

Clinical trials of myostatin blockade against muscular dystrophy and sarcopenia are now ongoing [34]. Thus, it is important to elucidate the role of the Dlk1-Dio3 locus in the muscle hypertrophy induced by myostatin blockade in postnatal and adult skeletal muscle.

## MATERIALS AND METHODS

### Animals

All animal experiments were conducted with approval from the Institutional Animal Care and Use Committee of Fujita Health University. Myostatin knockout mice previously described [3] were a kind gift from Dr. S.-J. Lee. As a control, age-matched C57BL/6 mice purchased from the Japan SLC, Inc. were used.

### Cell culture, immunofluorescence, and quantification of myotube diameters

Mouse C2C12 myoblasts were grown in Dulbecco's modified Eagle medium (DMEM) supplemented with 10% fetal bovine serum at 37°C and 5% CO_2_. Confluent C2C12 myoblasts were differentiated into myotubes using DMEM supplemented with 2% horse serum. Myotubes were transfected with 25 nM of miRNA mimics (miCENTURY OX miNaturals, Cosmo Bio) using Lipofectamine 2000 (Thermo Fischer Scientific) at 5 days after induction of differentiation. As a control, 25 nM of miRNA control (Non-target RNA, Cosmo Bio) was transfected. Two days after transfection, the myotubes were fixed and stained with an anti-myosin heavy chain antibody (MF20, Developmental Studies Hybridoma Bank) and DAPI. Cell images were visualized and myotube diameters were measured as described previously [12].

### RNA isolation, reverse transcriptase reaction, and quantitative-PCR

RNA samples were prepared from skeletal muscle as described previously [12]. In brief, the tibialis anterior muscles were surgically dissected from 5-, 9-, and 13-week-old mice under anesthesia and were quickly frozen in liquid nitrogen. Total RNAs were prepared using a miRNeasy Mini kit (Qiagen) according to the manufacturer's instructions.

The reverse transcriptase reaction was performed using a PrimeScript RT-PCR kit (TaKaRa) and a miScript Reverse Transcription Kit (Qiagen) for the detection of mRNAs and mature miRNAs, respectively. A SuperScript III First-Strand Synthesis System with Random primers (Thermo Fisher Scientific) was used for the detection of IG-DMR and IPW ncRNA according to the manufacturer's instructions.

The expression of mRNAs and ncRNAs was estimated by quantitative real-time PCR using a SYBR Premix Ex Taq (TaKaRa), and that of mature miRNAs were quantified by a miScript SYBR Green PCR Kit (Qiagen).

### Strand-specific RT-PCR

A strand-specific reverse transcriptase reaction was performed for detecting the paternally expressed *Rtl1* transcript and the maternally expressed *Rtl1as* transcript using a SuperScript III First-Strand Synthesis System using the previously reported RT-primer [35], *Rtl1* primer 5′- GGAGCCACTTCATGCCTAAGACGA-3′ and *Rtl1as* primer is 5′-GTGGAGAACTTCGCTGTCATCGC-3′. Semi-quantitative PCR for both strand-derived transcripts was conducted using TaKaRa Ex Taq (TaKaRa). The PCR results were quantified with the Image J 1.49v software, and the results were normalized to *Rpl26* expression. The primers used are listed in Supplementary Table S1.

### Genomic DNA isolation and bisulfite sequencing

Genomic DNA was isolated from the tibialis anterior muscles of 13-week-old mice using PureLink Genomic DNA Kits (Thermo Fisher Scientific). Genomic DNA (200 ng) was bisulfite treated using a Cells-to-CpG Bisulfite Conversion Kit (Thermo Fisher Scientific) according to the manufacturer's instructions. The PCR reaction was performed with an EpiTaq HS (TaKaRa) under the following conditions: denaturation at 94°C for 60 s and 35 cycles each of 94°C for 30 s, 55°C for 30 s, and 72°C for 60 s. A second nested PCR reaction was performed using the following conditions: denaturation at 94°C for 60 s and 25 cycles each of 94°C for 30 s, 55°C for 30 s, and 72°C for 60 s. The primers used are listed in Supplementary Table S1. The PCR products were subcloned into the pGEM T-easy vector (Promega), and clones derived from each independent subject (*n* = 3) were sequenced. The methylation status of the region (higher than 97% sequence identity) was determined and analyzed with QUMA (http://quma.cdb.riken.jp/top/quma_main_j.html).

## SUPPLEMENTARY MATERIALS TABLE


