# Supplementary material for: Myostatin-deficiency in mice increases global gene expression at the Dlk1-Dio3 locus in the skeletal muscle
Source: Oncotarget. 2016 Dec 15;8(4):5943–53. doi: 10.18632/oncotarget.13966 (PMC5351603; doi:10.18632/oncotarget.13966)
Supplement: Supplementary file 1 [file oncotarget-08-5943-s001.pdf]

Myostatin-deficiency in mice increases global gene expression at the Dlk1-Dio3 locus in the skeletal muscle

Supplementary Materials

Supplementary Table S1: Primer sequences used for PCR

Gene name	Sequence	
miR-411	TAGTAGACCGTATAGCGTAC	
miR-434-3p	TTTGAACCATCACTCGACTCC	
miR-193	AACTGGCCTACAAAGTCCCAG	
miR-379	TGGTAGACTATGGAACGTAG	
miR-193b	AACTGGCCCACAAAGTCCCG	
miR-22	miScript Primer Assay (Qiagen, MS00001330)	
miR-223	miScript Primer Assay (Qiagen, MS00001960)	
U6	miScript Primer Assay (Qiagen)	
miR-127	TCGGATCCGTCTGAGCTTGG	
miR-300	TATGCAAGGGCAAGCTCTCTT	
miR-329	AACACACCCAGCTAACCTTT	
miR-337	miScript Primer Assay (Qiagen, MS00011844)	
miR-376a	ATCGTAGAGGAAAATCCACG	
miR-381	TATACAAGGGCAAGCTCTCTG	
miR-434-5p	GCTCGACTCATGGTTTGAAC	
miR-540-3p	AGGTCAGAGGTCGATCCTG	
miR-543-3p	AAACATTCGCGGTGCACTTCT	
miR-486	TCCTGTACTGAGCTGCCCCG	
	**Forward**	**Reverse**
pri-miR-127	CTGGCTTTCTCTTGCATCCT	CGGATCCGATGATCTTTCTG
pri-miR-411	ACACGGTCCACTAACCCTCA	ATCATGGAGAAGCCCTGATG
Dlk1	ACTTGCGTGGACCTGGAGAA	CTGTTGGTTGCGGCTACGAT
Gtl2	TTGCACATTTCCTGTGGGAC	AAGCACCATGAGCCACTAGG
Rian	TCGAGACACAAGAGGACTGC	ATTGGAAGTCTGAGCCATGG
Mirg	CCTTCCTGGATCTCTCGCTT	GTGGGAGTTGAAACATGGGT
Rtl1	TACTGCTCTTGGTGAGAGTGGACCC	GGAGCCACTTCATGCCTAAGACGA
Rtl1as	TCTCCACTCGAGGGTACTCCACCT	GTGGAGAACTTCGCTGTCATCGC
IGF2	TCAGCCAAGCATGGCACAG	TACCAGGCCAATTCATAGTCTCCAA
Rpl26	GGTCTATGCCCATTCGGAAGG	TCGTTCGATGTAGATGACGTACT
IG-DMR ncRNA	AGAAGCTGTGGTGGGATTGCT	AGGGCCACTTGCATCAGAAT
IPW lncRNA	TGCCTGAGGAAAAGCGAGTT	GTGGTGTCTGCTGCTACACA
Sfrp1	TGGCCCGAGATGCTCAAATG	GGTTGTACCTTGGGGCTTAGA
Sfrp2	CGTGGGCTCTTCCTCTTCG	ATGTTCTGGTACTCGATGCCG
Sfrp4	AGAAGGTCCATACAGTGGGAAG	GTTACTGCGACTGGTGCGA
Sfrp5	CACTGCCACAAGTTCCCCC	TCTGTTCCATGAGGCCATCAG
Dnmt3a1	GAGGGAACTGAGACCCCAC	CTGGAAGGTGAGTCTTGGCA
Dnmt3a2	CTCACACCTGAGCTGTACTGCAGAG	CTCCACCTTCTGAGACTCTCCAGAG
IG-DMR for Bisulfite Sequencing (1st)	TAAGTGTTGTGGTTTGTTATGGGTA	CCATCCCCTATACTCAAAACATTCT
IG-DMR for Bisulfite Sequencing (2nd)	TGGTTTGTTATGGGTAAGTTTTATG	CTTCCCTCACTCCAAAAATTAAAA
Gtl2-DMR for Bisulfite Sequencing (1st)	TTTTTTGTTGTAGTTTGGGTG	CCATATCTCTCACCATTAATA
Gtl2-DMR for Bisulfite Sequencing (2nd)	GTTATAGTAATTTGTTATAGAATTTGGGG	AAACTTTCAACCACCAAAACC
